# Rotigotine transdermal patch in Parkinson’s disease: a narrative review of pharmacology, clinical evidence, special-population management, and health economics

**DOI:** 10.3389/fphar.2026.1933610

**Published:** 2026-10-06

**Authors:** Xuelin Sun, Dongfang Qian, Wenjing Zhao, Jingyi Zhang, Xin Hu, Yatong Zhang

**Affiliations:** 1 Department of Pharmacy, Beijing Hospital, National Center for Gerontology, National Clinical Research Center for Gerontology, The Key Laboratory of Geriatrics of NHC, Institute of Geriatric Medicine, Chinese Academy of Medical Sciences, Beijing, China; 2 Beijing North Medical Health Economic Research Center, Beijing, China; 3 College of Business Administration, Shenyang Pharmaceutical University, Shenyang, China

**Keywords:** continuous dopaminergic stimulation, health economics, narrative review, Parkinson’s disease, rotigotine, special populations, transdermal patch

## Abstract

**Background:**

Parkinson’s disease (PD) requires lifelong dopaminergic therapy, yet oral routes are frequently compromised by gastrointestinal dysfunction, dysphagia, and perioperative fasting. Abrupt withdrawal of oral dopaminergic drugs may precipitate severe motor deterioration or parkinsonism-hyperpyrexia syndrome. Rotigotine transdermal patch delivers 24-h continuous dopaminergic stimulation (CDS) via a non-ergot dopamine agonist (DA) formulation that bypasses first-pass metabolism and gastrointestinal absorption, and may be particularly useful when oral dopaminergic delivery becomes unreliable or is not possible.

**Objective:**

To synthesize published evidence on rotigotine pharmacology, clinical efficacy, management in vulnerable populations, safety, real-world effectiveness, and health economics, with emphasis on the incremental contribution of a scenario-based, Asia/China-inclusive, and pharmacy-oriented narrative relative to prior general reviews.

**Methods:**

Narrative review of randomized controlled trials, meta-analyses, regulatory documents, pharmacoeconomic models, and prospective observational studies identified through structured literature search and bibliographic screening.

**Results:**

Transdermal bioavailability is approximately 37% versus less than 5% for oral rotigotine. Pivotal Chinese trials SP0914 (early PD: UPDRS II + III −4.9 vs. −0.2) and SP1037 (advanced PD: placebo-adjusted off-time reduction −1.20 h/d [95% CI −1.83 to −0.57]; within-group change −2.36 h/d) demonstrated efficacy; meta-analyses support motor and selected non-motor benefits, including sleep, mood, and gastrointestinal tolerability, whereas swallowing-related signals remain preliminary and based mainly on small observational studies. Perioperative fasting, dysphagia, end-stage disease, and hepatorenal impairment represent clinically important scenarios in which non-oral delivery may be considered, although supporting evidence outside pivotal motor trials is often limited. Application-site reactions (ASRs) are the principal local adverse events; structured 14-day site rotation reduced ASR from 50.0% to 20.3% (P = 0.0373). Chinese cost-effectiveness analyses reported an incremental cost-effectiveness ratio of 1,118,617.81 CNY per quality-adjusted life year versus pramipexole immediate-release at market price, with price-sensitivity thresholds of 28.5% and 9.5% reductions.

**Conclusion:**

Rotigotine provides CDS for patients with PD when oral delivery is unreliable; individualized patient selection, structured pharmaceutical care, and updated pharmacoeconomic evaluation are warranted.

## 1 Introduction

Rotigotine, chemically (6S)-6-{propyl [2-(2-thienyl)ethyl]amino}-5,6,7,8-tetrahydro-1-naphthol, is the (−)-enantiomer of an aminotetrahydronaphthalene derivative (Jang and Jeong, 2025; Waters, 2013; Frampton, 2019). Its molecular spatial conformation closely resembles endogenous dopamine, providing a structural basis for broad receptor affinity and pharmacological activity in Parkinson’s disease (PD) (Scheller et al., 2005; Wood et al., 2015). As a major non-ergot dopamine agonist (DA) in contemporary PD pharmacotherapy, rotigotine combines distinct pharmacology with a transdermal drug delivery system (TDDS). Its most distinctive clinical added value relative to oral dopaminergic therapies is route independence: continuous delivery that can be maintained when gastrointestinal absorption is impaired or oral intake is interrupted (Ikeda et al., 2016; Frampton, 2019). Pharmacologically, rotigotine may confer advantages in bradykinesia and postural-gait disturbance through sustained dopaminergic receptor activation rather than intermittent oral peaks.

A defining characteristic of the rotigotine patch is formulation innovation. The TDDS achieves continuous dopaminergic stimulation (CDS) over 24 h (Waters, 2013; Frampton, 2019). Compared with oral therapy, transdermal delivery maintains relatively stable steady-state plasma concentrations, reducing peak-trough fluctuations that produce pulsatile dopamine receptor stimulation (Waters, 2013; Frampton, 2019); however, whether this theoretical PK advantage translates into a sustained reduction in long-term motor fluctuations or dyskinesia remains uncertain and requires confirmation in long-term comparative studies. Pharmacokinetically, transdermal administration bypasses gastrointestinal absorption and first-pass hepatic metabolism (Waters, 2013; Frampton, 2019). Because oral rotigotine bioavailability is below 5%, transdermal absolute bioavailability rises to approximately 37%, supporting stable systemic entry and central nervous system penetration across the blood-brain barrier. This route does not depend on gastrointestinal function and therefore holds particular value in PD patients with dysphagia, delayed gastric emptying, or perioperative nil per os (NPO) status.

Rotigotine transdermal patch (Neupro and regional branded formulations) is approved in numerous countries with substantial clinical experience. It received European Union approval in 2006 and China National Medical Products Administration (NMPA) import approval in June 2018. Indications span the PD disease course: monotherapy in early idiopathic PD and combination with levodopa in mid-to-advanced disease, especially when wearing-off or on-off motor fluctuations limit oral regimens. Relative to conventional oral dopamine agonists, the transdermal formulation provides pharmacokinetic continuity and route independence that may preserve dopaminergic treatment when gastrointestinal absorption is impaired or oral intake is interrupted (Center for Drug Evaluation, NMPA, 2022; Dharmian et al., 2025).

This narrative review synthesizes published evidence on rotigotine pharmacology, formulation science, clinical efficacy across motor and non-motor domains, management in clinically vulnerable populations, safety, health economics, and real-world effectiveness. The unifying clinical thread of this review is that transdermal rotigotine is most useful when oral dopaminergic therapy becomes unreliable or is not possible. Particular emphasis is therefore placed on perioperative fasting, dysphagia with pharyngeal drug retention, gastroparesis, intestinal bacterial metabolism of levodopa, end-stage inability to swallow, and age-related organ impairment (Table 1). Outside these route-limited contexts, oral dopamine agonists and levodopa remain foundational, and rotigotine should be positioned as a complementary rather than universally preferential option. Scenario-based analysis aligns with contemporary PD management goals that emphasize continuous, individualized, and caregiver-aware therapy rather than reflexive oral dose escalation alone.

**TABLE 1:** Clinical situations in which transdermal rotigotine may offer an advantage over oral dopaminergic therapy.

Clinical situation	Why oral therapy may fail	Potential advantage of transdermal rotigotine	Evidence strength
Perioperative NPO/fasting	Missed oral doses; withdrawal risk including PHS	Maintains dopaminergic exposure without oral intake	Limited observational/practice series
Dysphagia/pharyngeal retention	Incomplete swallowing; delayed-on	Bypasses oral route; possible swallow-related signals	Small VF/FEES studies; preliminary
Gastroparesis/SIBO/gut metabolism of levodopa	Erratic intestinal absorption	Absorption independent of GI transit/microbiota	Mechanistic + switch/RWE support
End-stage/fluctuating consciousness	Unsafe or unreliable oral administration	Once-daily visible dosing; nursing verification	Small observational cohorts; caution for DA toxicity
Hepatic/renal comorbidity with complex oral regimens	Unpredictable oral PK; polypharmacy	Labeling supports limited dose adjustment needs for parent drug	PK studies + labeling; clinical caution remains

Previous reviews of rotigotine, including the widely cited CNS Drugs appraisal by Frampton (2019) and related product- or symptom-focused overviews, have comprehensively summarized pharmacokinetics, pivotal efficacy for motor symptoms, selected non-motor outcomes, and overall tolerability of the transdermal patch. The present narrative review is not intended to duplicate that general clinical overview. Instead, it adds three layers of novel, practice-oriented synthesis. First, it organizes evidence around a single clinical question—when oral dopaminergic therapy becomes unreliable or is not possible—and provides an explicit scenario map (Table 1) spanning perioperative nil per os status, dysphagia with pharyngeal drug retention, gastroparesis and gut-related levodopa failure, end-stage care, and hepatorenal impairment, with claims moderated by evidence strength (Table 2). Second, it integrates China- and Asia-specific pivotal, regulatory, real-world, and pharmacoeconomic evidence (including Chinese SP0914/SP1037 trials, NMPA/CDE labeling context, Asian observational cohorts, and Chinese cost-effectiveness analyses) that is only sparsely covered in earlier Western-centered reviews. Third, it emphasizes pharmacy-facing operational content—approximate dose-conversion references, application-site reaction prevention, and interpretation of health-economic model assumptions—needed for multidisciplinary medication management beyond a conventional efficacy-and-safety narrative (Frampton, 2019).

**TABLE 2:** Selected clinical evidence for rotigotine, emphasizing design, main findings, and limitations.

Study/source	Design/N (approx.)	Main finding	Key limitation
SP512 (Watts et al., 2007)	RCT, early PD	UPDRS improvement vs. placebo as monotherapy	Early-PD population; older global cohort
SP0914 (Zhang et al., 2016)	RCT, Chinese early PD	UPDRS II + III −4.9 vs. −0.2 (placebo-adjusted contrast presented)	Regional population; ASR/withdrawal considerations
SP1037 (Zhang et al., 2017)	RCT, Chinese advanced PD	Off-time LS mean −1.20 h/d vs. placebo (95% CI −1.83 to −0.57)	Do not equate with −2.36 h/d within-group change
Hirano/Fukae swallow studies	Small VF/FEES observational	Signals for transit/PAS/retention-related delayed-on	Preliminary; not definitive clinical indication evidence
Asian RWE (Zhu et al., 2025; Ito et al., 2022)	Prospective/PMS cohorts	Effectiveness and tolerability at lower mean doses	Confounding; non-randomized

## 2 Methods

A structured literature search was conducted across international and Chinese databases, including PubMed, Embase, Web of Science, CNKI (China National Knowledge Infrastructure), and Wanfang Data. The search covered the period from database inception to February 2026 using combinations of key words including “rotigotine,” “transdermal patch,” “Parkinson’s disease,” “continuous dopaminergic stimulation,” “dysphagia,” “perioperative,” “dose conversion,” “safety,” “adverse events,” “health economics,” “cost-effectiveness,” “pharmacokinetics,” “formulation,” “swallowing,” “end-stage,” “hepatic,” and “renal,” combined with Boolean operators and, where available, database-specific subject headings (e.g., MeSH terms). Eligible sources primarily included peer-reviewed randomized controlled trials, systematic reviews and meta-analyses, prospective and retrospective observational studies, case series and case reports, and clinical practice guidelines. To support regulatory, labeling, and Chinese pharmacoeconomic context, selected non-peer-reviewed sources were retained when they provided extractable technical or economic parameters not available elsewhere—specifically regulatory technical documents (e.g., China NMPA/CDE review reports and product labeling) and one master’s thesis underlying a published Chinese cost-effectiveness analysis. Conference abstracts without full-text availability and sources lacking extractable clinical, pharmacokinetic, or economic data on rotigotine were excluded. Evidence was synthesized narratively with explicit attention to study design and evidence strength (RCT/meta-analysis versus observational/case-level data).

## 3 Mechanism of action

The principal pathological feature of PD is progressive degeneration and loss of dopaminergic neurons in the substantia nigra pars compacta, leading to striatal dopamine deficiency and the cardinal motor features of bradykinesia, resting tremor, rigidity, and postural-gait abnormality. Enhancing or substituting central dopaminergic neurotransmission therefore underpins pharmacological management. Conventional oral levodopa and short half-life DAs typically produce rapid absorption and clearance, generating pronounced plasma concentration swings described as pulsatile stimulation. Long-term pulsatile dopaminergic stimulation may induce striatal post-synaptic adaptations such as abnormal receptor sensitivity, signal transduction imbalance, and basal ganglia circuit remodeling, and these changes are associated with wearing-off, end-of-dose deterioration, and dyskinesia (Waters, 2013).

CDS has emerged as a therapeutic paradigm aiming to maintain relatively stable drug exposure and reduce non-physiological receptor activation from rapid concentration changes; whether this reliably lowers long-term motor-complication risk remains incompletely established in comparative longitudinal trials (Waters, 2013; Frampton, 2019). Rotigotine simultaneously agonizes dopamine D1, D2, D3, D4, and D5 receptor subtypes with a binding profile resembling endogenous dopamine (Scheller et al., 2005; Wood et al., 2015). Co-activation of D1/D2 pathways is thought to approximate physiological dopaminergic transmission and may benefit bradykinesia and gait disorders. Beyond classical dopaminergic activity, rotigotine exhibits 5-hydroxytryptamine receptor 1A (5-HT1A) receptor agonism and α2B adrenergic antagonism, mechanisms that have been hypothesized to contribute to the modulation of anxiety, depression, and dyskinesia. Relative to short-acting oral dopaminergic drugs, transdermal rotigotine sustains more uniform receptor stimulation across 24 h, which may improve nocturnal and early-morning motor symptoms while attenuating off-time non-motor fluctuations.

Preclinical and clinical literature consistently links pulsatile levodopa exposure to progressive shortening of levodopa response duration and emergence of peak-dose dyskinesia (LeWitt, 2015; Waters, 2013). Although rotigotine is not levodopa, its transdermal profile addresses the same underlying problem: non-physiological receptor occupancy patterns (Scheller et al., 2005). Physiologically based pharmacokinetic modeling of transdermal rotigotine supports predictable dermal absorption and systemic exposure interpretation, facilitating dose individualization in special populations where oral pharmacokinetics are unpredictable (Jang and Jeong, 2025). The combination of D1–D5 agonism with serotonergic and adrenergic modulation differentiates rotigotine from selective D2/D3 agonists and may explain observed benefits in mood, sleep, and certain motor subdomains such as gait freezing that respond incompletely to levodopa alone.

## 4 Formulation technology

The rotigotine patch is a technically demanding transdermal product whose development must simultaneously achieve effective dermal delivery and long-term physical stability (Oh et al., 2019). Rotigotine is highly lipophilic and exhibits low solubility in traditional silicone-based matrices (often below 3%) (Liu et al., 2024). Yet adequate transdermal driving force requires a relatively supersaturated formulation state. Although supersaturation enhances flux, it is thermodynamically unstable and predisposes to crystallization, which can alter release kinetics and bioavailability. Maintaining stability at high drug loading represents a core engineering challenge.

In 2008, global recall in Europe and North America followed observation of snowflake-like crystals within patches. Subsequent investigations attributed the phenomenon primarily to transition from metastable polymorph I to thermodynamically more stable polymorph II, reducing release performance and clinical exposure. Polymorph control thus directly affects both efficacy and safety of transdermal rotigotine (Waters, 2013).

Current commercial patches predominantly employ a drug-in-adhesive (DIA) architecture in which active ingredient is dissolved or uniformly dispersed within a pressure-sensitive adhesive (Dharmian et al., 2025). Compared with reservoir designs, DIA systems are thinner, more flexible, and less prone to dose dumping (Liu et al., 2024; Oh et al., 2019). Drug forms a dynamic dispersion within the polymer matrix and diffuses toward the skin under concentration-gradient drive, yielding relatively stable release (Oh et al., 2019). Typical laminate structure includes backing film, drug-containing adhesive, and protective liner; coordinated layer design stabilizes release and transdermal delivery. To stabilize supersaturated systems, crystal-inhibition strategies employ polymers and functional excipients to suppress nucleation and growth. Soluplus and TPGS synergistic systems reduce crystallization tendency through hydrogen bonding and steric hindrance; molecular modeling indicates favorable binding free energy maintaining amorphous or high-energy dispersed states. Polyvinylpyrrolidone (PVP) may form mesh-like structures that physically block crystal growth. Oleic acid serves both as a permeation enhancer by modulating stratum corneum lipid arrangement and as an extender of the metastable zone, thereby delaying crystallization. Collectively, these excipient and process innovations improve storage stability, reduce transdermal resistance, shorten lag time, and support the relatively flat exposure profile that underpins CDS in clinical use.

Regulatory technical review documents from China’s Center for Drug Evaluation describe quality control parameters for rotigotine patch manufacturing, including adhesive rheology, residual solvent limits, and *in vitro* release specifications that evolved after the 2008 recall experience. Smart drug delivery reviews emphasize that successful transdermal PD therapy requires integration of material science, clinical pharmacology, and patient education rather than isolated efficacy endpoints. For hospital formulary committees and clinical pharmacists, understanding formulation complexity justifies structured training on application technique, storage conditions, and disposal of used patches. Improper storage at elevated temperature can accelerate polymorphic transition and compromise the very CDS advantage that underpins rotigotine’s therapeutic rationale (Center for Drug Evaluation, NMPA, 2022; Dharmian et al., 2025; Liu et al., 2024).

## 5 Clinical efficacy

Clinical efficacy evaluation of rotigotine must be interpreted within the broader PD therapeutic landscape comprising levodopa, monoamine oxidase B inhibitors, catechol-O-methyltransferase inhibitors, and multiple DA formulations with varying half-lives. Rotigotine does not replace levodopa in advanced disease but complements it when fluctuations persist, and may serve as monotherapy in early disease to defer levodopa-related motor complications. Available evidence is strongest for motor domains validated in randomized controlled trials (RCTs), directionally consistent but more heterogeneous for non-motor symptoms (NMS), and most clinically consequential—yet often supported only by observational or small mechanistic studies—in patient subsets in whom oral pharmacokinetics are inherently unreliable. Throughout this section we prioritize placebo-adjusted between-group effects for RCTs and explicitly flag lower-level evidence where relevant (Table 2) (Frampton, 2019; Watts et al., 2007; Zhang et al., 2020; de Bie et al., 2025).

### 5.1 Motor symptom improvement

Efficacy of rotigotine patch in PD motor symptom management is supported by multiple randomized controlled trials (RCTs), systematic reviews, and meta-analyses (Watts et al., 2007 Zhang et al., 2020). Pooled analyses demonstrate significant improvements in UPDRS part II (activities of daily living), part III (motor examination), and combined II + III scores, with early onset and durable maintenance (Zhang et al., 2020). Patients often show meaningful ADL gains by 8 weeks, with further motor improvement from 12 to 24 weeks of continued therapy (Zhang et al., 2016). In Chinese early PD patients (SP0914), 24-week treatment produced UPDRS II + III change of −4.9 versus −0.2 points versus placebo (Zhang et al., 2016). Response rates exceeded placebo across thresholds of ≥20%, ≥25%, and ≥30% improvement. In Chinese advanced PD with inadequate levodopa control (SP1037), adjunctive rotigotine reduced absolute daily off-time by a least-squares mean of 1.20 h versus placebo (95% CI −1.83 to −0.57; P = 0.0002); the corresponding within-group change from baseline was −2.36 h/day with rotigotine and −1.13 h/day with placebo, so the within-group figure should not be interpreted as the placebo-adjusted treatment effect (Zhang et al., 2017).

Beyond global motor scores, rotigotine shows value in early-morning off, nocturnal motor dysfunction, and freezing of gait (FOG) (Ikeda et al., 2016; Trenkwalder et al., 2011). Twenty-four-hour transdermal exposure provides CDS throughout the circadian cycle. The RECOVER study (SP889) reported significant improvement in UPDRS III upon morning awakening after rotigotine treatment (Trenkwalder et al., 2011). FOG-related studies noted reduced off-time FOG scores, potentially reflecting sustained exposure and broad receptor agonism (Ikeda et al., 2016). Large Japanese and Chinese real-world cohorts indicate symptom improvement in approximately 70% of patients, including those aged 80 years and older. The 2017 United Kingdom National Institute for Health and Care Excellence (NICE) guideline recommends considering rotigotine when levodopa or oral DAs inadequately control nocturnal motor symptoms.

Global early PD evidence from SP512 established proof of concept for transdermal rotigotine as monotherapy, with UPDRS improvements and acceptable tolerability that informed subsequent regional development including Chinese SP0914 (Watts et al., 2007; Zhang et al., 2016). The Chinese meta-analysis pooled RCT data confirming directional consistency across UPDRS domains and safety outcomes, strengthening level 1a evidence for motor benefit in Asian populations (Zhang et al., 2020). When interpreting motor outcomes, clinicians should distinguish statistically significant group differences from clinically meaningful change; a −4.9-point UPDRS II + III shift in SP0914 exceeds commonly cited minimal clinically important difference thresholds for early PD trials. In SP1037, the placebo-adjusted off-time reduction of 1.20 h per day (rather than the 2.36-h within-group change) is the appropriate between-group estimate of benefit and remains clinically meaningful for self-care, mobility, and social participation (Zhang et al., 2017). Consistent with this magnitude, a recent International Parkinson and Movement Disorder Society (MDS) evidence-based medicine review reported placebo-adjusted OFF-time reductions with rotigotine of approximately 0.9–1.8 h/day across randomized trials and classified rotigotine as efficacious for motor fluctuations (de Bie et al., 2025). Titration schedules in pivotal trials typically employed weekly dose escalation over several weeks; premature discontinuation during early nausea-prone titration remains a common real-world failure mode that pharmacists can mitigate through anticipatory counseling and antiemetic strategies when appropriate.

### 5.2 Non-motor symptom improvement

PD burden extends beyond motor disability (Frampton, 2019; Nomoto et al., 2024). NMS—sleep disturbance, swallowing dysfunction, gastrointestinal symptoms, anxiety, depression, and autonomic dysfunction—occur throughout the disease course and may impair quality of life and caregiver burden as profoundly as motor signs (Fanciulli et al., 2013; Nomoto et al., 2024; Sieb et al., 2015; Zhu et al., 2025). NMS management is integral to comprehensive PD care. Evidence for rotigotine in NMS is expanding across RCTs, real-world studies, and systematic reviews (Ito et al., 2022; Trenkwalder et al., 2011; Zhang et al., 2020; Zhu et al., 2025). Plausible mechanisms include CDS reducing exposure fluctuation, broad D1–D5 agonism, and modulation of non-dopaminergic targets such as 5-HT1A receptors.

#### 5.2.1 Sleep-related symptoms

A meta-analysis of 11 RCTs reported improvements in Parkinson’s Disease Sleep Scale (PDSS/PDSS-2) and Non-Motor Symptoms Scale (NMSS) scores with rotigotine, indicating benefits in sleep-related and broader non-motor domains (Zhang et al., 2020). Video-polysomnography (VPSG) studies demonstrated reduced nocturnal awakenings, increased sleep efficiency, and shortened sleep latency (Wang et al., 2017). Objectively, sleep efficiency rose from 68.59% to 74.04% (P = 0.034), sleep latency decreased, and periodic leg movement index fell from 22.68 to 14.24 (P < 0.001), suggesting improvements in sleep architecture and nocturnal motor phenomena (Wang et al., 2017). RECOVER additionally linked rotigotine to PDSS-2 improvement alongside early-morning motor gains (Trenkwalder et al., 2011).

Sleep dysfunction in PD encompasses insomnia, REM sleep behavior disorder, nocturia-related fragmentation, and nocturnal motor phenomena including akinesia and painful cramps (Zhang et al., 2020). Because sleep disruption reciprocally worsens daytime motor and cognitive performance, therapies that simultaneously address nocturnal motor symptoms and sleep architecture may yield disproportionate daytime benefit (Wang et al., 2017). Rotigotine’s 24-h profile contrasts with bedtime-only oral adjuncts that leave early-morning intervals undertreated. NICE guidance specifically elevates rotigotine when oral regimens fail nocturnal motor control, reflecting regulatory recognition of this unmet need (National Institute for Health and Care Excellence, 2017). Clinicians should pair subjective sleep scales with motor diaries when evaluating rotigotine response, as patients may report improved morning mobility before perceiving sleep quality change.

#### 5.2.2 Swallowing and sialorrhea

Dysphagia increases aspiration pneumonia, hospitalization, and mortality risk in PD. In a small Japanese videofluoroscopic study, low-dose rotigotine (2 mg/24 h) was associated with shortened pharyngeal transit time, improved oral-pharyngeal coordination, and improved penetration-aspiration scale (PAS) safety scores; these findings are preliminary and require confirmation. Fiberoptic endoscopic evaluation of swallowing (FEES) demonstrated reduced pooling of saliva, suggesting benefit for sialorrhea and swallow-related secretion management. These findings complement motor gains by addressing a high-impact NMS domain with direct safety implications (Fukae et al., 2020; Hirano et al., 2019; Kim et al., 2023; Schirinzi et al., 2017).

The relationship between swallowing dysfunction and oral medication failure creates a vicious cycle: worsening motor control further impairs swallow safety, while unreliable oral absorption prevents effective motor treatment (Kim et al., 2023; Schirinzi et al., 2017). Scoping reviews of advanced PD without safe oral access emphasize non-oral dopaminergic options as potentially important palliative and acute-care considerations when oral access is unsafe (Kim et al., 2023). Rotigotine does not replace speech-language pathology or dysphagia rehabilitation but may stabilize motor substrates of swallow coordination while eliminating dependence on oral delivery (Fukae et al., 2020). Salivary pooling and drooling additionally carry psychosocial stigma; preliminary drooling studies suggest rotigotine may reduce visible sialorrhea, though confirmatory trials remain limited. Fiberoptic documentation of reduced pharyngeal residue supports mechanistic plausibility beyond subjective patient report.

#### 5.2.3 Gastrointestinal symptoms and tolerability

The transdermal route of rotigotine avoids gastrointestinal absorption and first-pass metabolism, providing a pharmacological rationale for its favorable gastrointestinal tolerability. A prospective multicenter switch study reported that PD patients converting from oral DAs to rotigotine experienced reduced frequency and severity of nausea, bloating, and heartburn; 86% expressed satisfaction with gastrointestinal symptom improvement. Improvement rates for nausea, heartburn, and bloating were 68.4%, 61.5%, and 54.1%, respectively. Chinese real-world data documented gastrointestinal adverse event rate of 4.0%, supporting favorable GI tolerability in routine practice (Van Kessel et al., 2017; Woitalla et al., 2015; Zhu et al., 2025).

Gastrointestinal adverse effects of oral DAs and levodopa—nausea, vomiting, bloating—often emerge during initiation and may persist in sensitive individuals, leading to treatment discontinuation despite motor benefit (Van Kessel et al., 2017). The prospective switch trial by Woitalla and colleagues provides direct patient-reported evidence that transdermal conversion improves tolerability independent of motor endpoints (Woitalla et al., 2015). Preclinical work on PD medications altering small-intestinal motility and microbiota composition contextualizes why oral route reliability may deteriorate even when gastric emptying appears clinically silent (Van Kessel et al., 2017). For patients with concurrent irritable bowel symptoms or prior DA-induced nausea, rotigotine represents a route-of-administration solution rather than merely a different molecule within the same oral tolerability class.

#### 5.2.4 Mood and non-motor fluctuations

Psychiatric symptoms and non-motor fluctuations substantially affect QoL. Rotigotine’s full receptor agonist profile and non-dopaminergic activity provide pharmacological rationale for mood-domain benefit. *In vitro*, rotigotine potently activates D1–D5 receptors and 5-HT1A receptors (Ki approximately 30 nM), enriched in frontal cortical regions governing emotional regulation. Meta-analyses consistently report improvement in Beck Depression Inventory-II (BDI-II) scores and NMSS mood/affect subdomains. CDS maintained by 24-h patch delivery may attenuate off-time anxiety, apathy, and low mood linked to fluctuating receptor stimulation. Chinese real-world PDQ-8 data showed sustained gains in the emotional wellbeing dimension, corroborating trial-level neuropsychiatric findings (Scheller et al., 2005; Wang et al., 2018; Zhang et al., 2020; Zhu et al., 2025).

Non-motor fluctuations—including off-time anxiety, panic sensations, dysphoria, and cognitive slowing—may be as disabling as motor off-times yet are underdocumented in standard motor diaries (Wang et al., 2018; Zhang et al., 2020). Prescription trend analyses in Japanese advanced PD populations highlight growing clinician attention to NMS burden when selecting dopaminergic therapies (Nomoto et al., 2024). Case-level reports describe rotigotine benefit for anxiety during off-time in PD with dementia, though such evidence is anecdotal and requires cautious generalization (Fanciulli et al., 2013). Meta-analytic mood benefits should be interpreted alongside baseline psychiatric comorbidity and concurrent antidepressant use. Nevertheless, the pharmacological rationale linking 5-HT1A agonism to anxiolytic and antidepressant effects is well established in neuropsychopharmacology, and CDS may reduce trigger frequency for off-time mood deterioration.

#### 5.2.5 Daytime somnolence

Daytime sleepiness is a common DA-related adverse effect with heterogeneous impact across agents and patients. Meta-analytic comparison of rotigotine versus placebo showed no significant difference in somnolence incidence (relative risk 1.07; 95% CI 0.68–1.69; P = 0.77), suggesting rotigotine does not materially increase sleepiness risk relative to placebo. Among patients with prior oral DA-associated excessive daytime sleepiness (Epworth Sleepiness Scale [ESS] ≥10), Korean switch data indicated ESS decline after conversion to rotigotine; five of six patients with baseline hypersomnolence no longer met criteria post-switch. Open-label studies reported no worsening of daytime sleepiness at doses up to 8 mg/24 h, with ESS improvements in patients converted from other non-ergot DAs. However, SP0914 noted ESS increase of 1.0 versus 0.2 in placebo, and a Chinese VPSG study observed ESS rise from 4.72 to 6.24 (P = 0.077), underscoring individual variability and need for clinical monitoring (Kim et al., 2011; Ohta and Osada, 2015; Wang et al., 2017; Zhang et al., 2016; Zhang et al., 2020).

Regulatory labeling and prescriber information recommend monitoring daytime sleepiness during dose escalation and advising patients about driving restrictions if sudden sleep episodes occur (Zhang et al., 2020). The overall balance of somnolence evidence is mixed rather than definitive: meta-analytic incidence versus placebo was neutral, yet individual trials and open-label switch series show bidirectional ESS changes. Rotigotine may be considered as a switch option in selected hypersomnolent patients failing oral therapy, but sudden-onset sleep episodes remain a class warning and require individualized monitoring (Ohta and Osada, 2015; Zhang et al., 2020). Clinicians should document baseline ESS before and after conversion to objectify change (Wang et al., 2017).

### 5.3 Quality of life

Quality of life (QoL) integrates motor and non-motor relief, treatment adherence, and psychosocial participation. Rotigotine-associated QoL gains likely reflect concurrent symptom improvement, CDS-related stability, and simplified once-daily transdermal administration. Chinese real-world evidence in 803 patients showed PDQ-8 total score improvement averaging 7.34 points at 12 weeks. Advanced patients improved more than early-stage patients (−8.16 vs. −6.87 points). Patient Global Impression of Change (PGIC) data aligned: 79.9% reported overall improvement at 12 weeks without documented severe worsening during follow-up (Zhu et al., 2025).

Improved activities of daily living contribute directly to QoL. Meta-analyses and pivotal trials including SP512 and SP0914 demonstrate greater UPDRS II improvement versus placebo (P < 0.00001). Japanese real-world observations noted gains in facial expression, sit-to-stand, gait, postural stability, and bradykinesia—functions central to independence. Beyond symptom scales, treatment experience matters: 46% of patients in a Korean conversion study preferred patch therapy, citing once-daily convenience (72%), lower interference with daily activities (54%), and reduced need to track dosing times (54%). Chinese data reported near-99% adherence and 79.9% PGIC improvement. German CARE-ACT showed high caregiver and physician acceptance, particularly when cognitive impairment or dysphagia complicate oral regimens; visible patch status simplifies supervision and may reduce caregiver burden. Collectively, rotigotine is associated with improvements in quality of life spanning physiological function, emotional health, and social participation (Zhu et al., 2025).

Patient-reported outcome instruments such as PDQ-8 capture stigma, social isolation, and fear of dependency that physician-rated scales omit (Watts et al., 2007; Zhang et al., 2016; Zhang et al., 2020). The larger QoL gains observed in advanced versus early PD in Chinese RWE may reflect greater baseline symptom burden and therefore larger absolute room for improvement, rather than lesser benefit in early disease (Ito et al., 2022). RECOVER’s dual endpoints—PDSS-2 and morning UPDRS III—illustrate how rotigotine may address linked nocturnal and early-morning symptom clusters that degrade QoL disproportionately. Medication adherence near 99% in Chinese real-world practice exceeds typical oral DA persistence reported in chronic disease literature, suggesting that formulation convenience translates into measurable behavioral outcomes. Caregiver-facing benefits documented in CARE-ACT should be weighed in holistic value assessments, particularly where informal caregiving hours represent a major societal cost.

## 6 Special-population management

Transdermal rotigotine bypasses gastrointestinal absorption and sustains relatively stable exposure, creating differentiated value in perioperative management, dysphagia, gastrointestinal dysfunction, end-stage disease, and altered hepatorenal function. Rotigotine undergoes extensive conjugative metabolism and renal/fecal elimination of metabolites; hepatic and renal impairment have limited impact on parent drug exposure within studied ranges (Cawello et al., 2014; Jang and Jeong, 2025; UCB Pharma, 2021; Waters, 2013). At the same time, patients who most need non-oral delivery—advanced age, multimorbidity, cognitive impairment, or end-stage disease—are also those at highest risk of dopamine-agonist toxicity (hallucinations/psychosis, impulse-control disorders, orthostatic hypotension, somnolence/sleep attacks, peripheral edema, and cognitive worsening). Accordingly, recommendations in this section are framed as conditional options for oral-route failure rather than routine substitutions for all advanced PD care.

Clinical pharmacists play a central role in special-population rotigotine use by documenting conversion rationale, verifying dose translation tables, educating on site rotation and heat avoidance, and coordinating perioperative medication reconciliation across neurology, anesthesia, and nursing services. Multidisciplinary pathways that embed these tasks reduce the risk of preventable harm from dopaminergic interruption or inappropriate antiemetic prescribing (Beckers et al., 2025; Geng et al., 2021; UCB Pharma, 2021).

### 6.1 Perioperative patients

Perioperative care is a critical medication management scenario for PD. Preoperative NPO requirements often interrupt oral anti-PD therapy, risking motor deterioration and complications. Abrupt dopaminergic withdrawal—especially levodopa—can precipitate severe bradykinesia, rigidity, and prolonged off-times; rarely, parkinsonism-hyperpyrexia syndrome (PHS) manifests with hyperthermia, autonomic instability, altered consciousness, and elevated creatine kinase. PD-related dysphagia further raises aspiration and pneumonia risk during hospitalization. Maintaining CDS perioperatively aims not only to stabilize symptoms but to prevent life-threatening withdrawal syndromes (Beckers et al., 2025; Factor and Santiago, 2020; Govindappa et al., 2015; Habibi et al., 2026; Hiramoto and Takahashi, 2024; Rosado et al., 2025).

The rotigotine patch can provide continuous dopaminergic delivery during fasting and may therefore offer a practical non-oral option in the perioperative setting. Pharmacokinetic data indicate steady state within 1–2 days of daily application, permitting flexible preoperative initiation or dose adjustment.

Perioperative conversion requires individualized dopaminergic translation (Beckers et al., 2025; Govindappa et al., 2015). Low-to-moderate dose patients may transition directly to rotigotine after oral cessation; high-dose regimens may need cross-titration retaining partial oral therapy while escalating patch dose to limit fluctuation (Waters, 2013). Reference conversions include rotigotine 16 mg/24 h ≈ 480 mg daily levodopa equivalent dose (LEDD; ∼30:1 ratio), pramipexole 1 mg ≈ rotigotine 4 mg, and ropinirole to rotigotine approximately 1.5:1—ratios with demonstrated feasibility in Asian overnight-switch cohorts (Kim et al., 2011; Chitnis et al., 2012). Pharmaceutical precautions include patch removal before MRI or cardioversion due to aluminum backing, avoidance of heating pads or direct heat over application sites, structured site rotation with ≥14-day interval before reusing the same area, and avoidance of dopamine antagonists such as metoclopramide for nausea or delirium because they may worsen PD symptoms.

Parkinsonism-hyperpyrexia syndrome resembles neuroleptic malignant syndrome and carries substantial mortality if dopaminergic therapy is not promptly restored. Case reports during general anesthesia, deep brain stimulation battery depletion, and pandemic-related care disruptions underscore that any scenario interrupting oral levodopa or DA delivery poses risk. Practice reviews recommend advance documentation of non-oral contingency plans. Rotigotine’s perioperative feasibility data, while derived from modest sample sizes, provide limited empirical support for guidelines advocating transdermal bridging. Multidisciplinary agreement—89% among both neurologists and anesthesiologists in published surveys—facilitates institutional protocol adoption. Electronic health record templates should capture patch dose, site, and scheduled change time alongside nil-by-mouth orders to prevent inadvertent polypharmacy or omission during care transitions. The approximate dose-conversion references and clinical considerations for these transitions are detailed in Table 3 (Beckers et al., 2025; Factor and Santiago, 2020; Govindappa et al., 2015; Habibi et al., 2026; Hiramoto and Takahashi, 2024; Korczyn et al., 2007; Fagerlund et al., 2013; Rosado et al., 2025; Yasutaka et al., 2020). Dose-conversion ratios are approximate reference values derived from clinical studies and/or consensus recommendations and should not be interpreted as universally applicable dose-equivalence rules. Dose conversion should be individualized according to clinical response, tolerability, local product labeling, and regional dosing recommendations, which may differ across countries.

**TABLE 3:** Approximate dose-conversion references for rotigotine transdermal patch.

Conversion scenario	Recommended dosing/conversion	Practical considerations/Key references
Rotigotine ↔ levodopa (LEDD)	16 mg/24 h ≈ 480 mg/d (∼30:1)	Individualize by symptom control and tolerability (Korczyn et al., 2007 ; Waters, 2013 )
Pramipexole → rotigotine	1 mg: 4 mg	Cross-titration over 2–4 weeks for high LEDD (Yasutaka et al., 2020 ; Chung et al., 2017 )
Ropinirole → rotigotine	∼1.5 : 1	Overnight switch feasible in Asian cohorts with monitoring ( Kim et al., 2011 )
Steady-state attainment	1–2 days of daily application	Apply before planned surgery or NPO period (Waters, 2013 ; UCB Pharma, 2021 )
Advanced PD typical dose	8 mg/24 h (max 16 mg/24 h)	Combine patch strengths (e.g., 6 + 4 mg) for titration (Zhang et al., 2017; UCB Pharma, 2021)

### 6.2 Dysphagia and gastrointestinal dysfunction

Swallowing impairment and gastroparesis are prevalent NMS that compromise oral drug delivery (Beckers et al., 2025; Geng et al., 2021). Tongue and pharyngeal hypomobility may retain medication in valleculae or pyriform sinuses; FEES studies linked pharyngeal drug retention to delayed-on phenomena (odds ratio 42.7), preventing timely small-intestinal absorption (Fukae et al., 2020). Levodopa effects on swallowing metrics remain inconsistent. Rotigotine patch circumvents the oral route: limited videofluoroscopic and FEES observations suggest possible improvements in pharyngeal transit, PAS scores, and salivary pooling, but the evidence base remains small and non-definitive.

Delayed gastric emptying affects 35%–100% of PD patients across disease stages (Doi et al., 2012). Oral levodopa absorption in the proximal small intestine is vulnerable to gastric retention, incomplete swallowing, and bacterial metabolism. Small intestinal bacterial overgrowth occurs in up to 55% of PD patients; *Enterococcus faecalis* tyrosine decarboxylase can degrade levodopa before absorption. For patients unable to use oral drugs reliably, rotigotine offers a non-oral alternative avoiding nasogastric complexity. Transdermal absorption with ∼37% bioavailability is independent of gastric emptying, intestinal absorption, and gut microbiota. Switch studies document GI symptom relief; Chinese RWE reports 4.0% GI adverse events, supporting tolerability when oral routes contribute to symptom burden (Beckers et al., 2025; Leta et al., 2023; Van Kessel et al., 2017; Van Kessel et al., 2019; Woitalla et al., 2015; Zhu et al., 2025).

European reviews of gastrointestinal barriers to levodopa transport synthesize evidence that gastroparesis, small-intestinal bacterial overgrowth, and protein-rich meal interactions collectively erode oral levodopa reliability across disease stages. Case reports document dramatic symptom fluctuations attributable to dysphagia alone, reinforcing that motor fluctuation workups should include swallow assessment when delayed-on or no-response phenomena occur despite adequate dosing. Rotigotine does not address constipation or primary gastroparesis pathophysiology but decouples dopaminergic efficacy from upper gastrointestinal transit variables. For patients requiring enteral feeding, transdermal rotigotine avoids compounding and tube-occlusion issues associated with crushed tablets or liquid formulations not formulated for enteral use (Doi et al., 2012; Fukae et al., 2020; Geng et al., 2021; Leta et al., 2023; Sato et al., 2018; Van Kessel et al., 2019).

### 6.3 End-stage Parkinson’s disease

Advanced PD (Hoehn and Yahr stages 3–5) features severe disability, motor complications, refractory NMS, and cognitive decline. Dopamine agonist use demands vigilance for neuropsychiatric toxicity; oral regimens often fail when consciousness fluctuates. Patient-centered end-stage planning should anticipate non-oral alternatives before crisis. Among 51 end-stage PD patients in the United Kingdom, 55% received rotigotine patch instead of oral therapy before death; this small, non-comparative observation suggests that continuous exposure is used in practice to maintain dopaminergic symptom control, but it does not establish comparative effectiveness or safety. Importantly, the same advanced/end-stage patients are particularly vulnerable to dopamine-agonist adverse effects—including hallucinations, psychosis, impulse-control disorders, orthostatic hypotension, somnolence, edema, and cognitive worsening—so slow titration, caregiver education, and a low threshold for dose reduction or discontinuation are essential. Per Chinese labeling, most fluctuating advanced patients respond at 8 mg/24 h with maximum 16 mg/24 h; combined patch strengths (e.g., 6 mg plus 4 mg) enable individualized titration. Stepwise dose escalation remains essential to limit psychosis. CARE-ACT reported caregiver and physician advantage scores of 1.32 and 1.46 (5-point scale), reflecting reduced burden from once-daily visible dosing. Hospitalized or palliative patients with multimorbidity (mean 4.6 comorbidities), cachexia, or poor peripheral perfusion may exhibit variable transdermal absorption and heightened sensitivity; dynamic clinical reassessment is required (Beckers et al., 2025; Ito et al., 2022; Kim et al., 2023; Fagerlund et al., 2013; Marchitto et al., 2019; Sieb et al., 2015).

Advance care planning in PD should discuss non-oral contingencies before emergency hospitalization renders shared decision-making impossible (Sato et al., 2018). Scoping reviews emphasize that patients without safe oral access require algorithmic escalation across transdermal, subcutaneous, and device-assisted levodopa options based on goals of care (Fukae et al., 2020). Rotigotine occupies a lower-complexity tier appropriate for home hospice and nursing facilities lacking apomorphine infusion expertise (LeWitt, 2015). Visible patch presence allows nursing staff to verify dosing without waking sedated patients for pill administration—a meaningful dignity consideration. However, cachexia and peripheral edema may alter skin adhesion and absorption; alternative sites and supplemental strategies should be anticipated. Psychosis risk from dopaminergic escalation in end-stage disease mandates slow titration and caregiver education on hallucination monitoring despite the convenience of patch delivery.

### 6.4 Hepatic and renal impairment

PD predominantly affects older adults with frequent hepatic or renal comorbidity and polypharmacy (Frampton, 2019; Nomoto et al., 2024). Hepatic impairment can destabilize oral drug bioavailability; renal dysfunction complicates dosing of renally cleared oral DAs such as pramipexole (Jenner et al., 2009; Frampton, 2019; Waters, 2013). Rotigotine is metabolized via sulfation, glucuronidation, and CYP-mediated N-dealkylation without dependence on a single CYP isoform. Mild-to-moderate hepatic impairment does not require dose adjustment; severe hepatic impairment lacks robust data and warrants caution with possible reduction if function worsens. Unbound rotigotine steady-state concentrations in mild-to-severe renal impairment do not exceed healthy volunteers; inactive conjugates and dealkylated metabolites may rise with renal decline without clinical effect. Phase III data and labeling support no routine dose adjustment in mild-to-severe renal impairment, including dialysis patients.

Polypharmacy in elderly PD patients frequently includes renally cleared agents requiring dose adjustment in chronic kidney disease. Simplifying the medication regimen by selecting rotigotine when DA therapy is indicated may reduce cumulative nephrotoxic psychotropic burden from accumulated pramipexole. Hepatic impairment studies demonstrate stable clearance in mild-to-moderate disease, supporting use in common age-related hepatic steatosis or mild enzyme elevation contexts when oral bioavailability would otherwise fluctuate unpredictably. Dialysis populations remain clinically relevant given cardiovascular comorbidity and hospitalization frequency in advanced PD; labeling support for no dose adjustment in dialysis provides practical reassurance, though clinical monitoring for orthostatic symptoms and neuropsychiatric effects remains mandatory (Cawello et al., 2014; Jang and Jeong, 2025; UCB Pharma, 2021; Waters, 2013).

## 7 Safety and tolerability

Safety assessment of rotigotine integrates trial-derived incidence rates, post-marketing surveillance, and pharmacovigilance case reports of rare but serious events. The transdermal route eliminates certain oral-specific adverse effects—esophageal irritation, gastric upset from local irritation—but introduces ASRs as a class effect of patch therapy. Dopaminergic systemic effects remain shared with oral DAs, including nausea, orthostatic symptoms, somnolence, and neuropsychiatric complications. Contextualizing trial adverse event rates against real-world data at lower mean doses is essential: Chinese RWE at 3.42 mg/24 h reports substantially lower overall adverse event rates than phase III trials titrating to higher targets, suggesting dose and population selection strongly modulate tolerability experience (Center for Drug Evaluation, NMPA, 2022; UCB Pharma, 2021; Zhang et al., 2016; Zhu et al., 2025).

### 7.1 Common adverse events

Safety of rotigotine patch is established in global multicenter trials, Chinese phase III studies, and large real-world cohorts. Transdermal delivery produces a distinct adverse-event profile combining application-site reactions (ASRs) and dopaminergic systemic effects. Two complementary safety reporting frames should not be conflated. Chinese product labeling reports a pooled any-adverse-event incidence of 72.5% with rotigotine versus 58.0% with placebo across Chinese phase III experience. By contrast, the individual pivotal publications report lower study-level rates: 57.3% versus 51.2% in early PD (SP0914) and 59.2% versus 50.0% in advanced PD (SP1037), reflecting trial-specific coding windows, populations, and concomitant therapy rather than contradictory findings. Common events in these programs included nausea, somnolence, erythema, and pruritus. Real-world rates are lower: Chinese 27-hospital study (803 patients) reported 17.6% overall adverse events with application-site pruritus 6.5%, dermatitis 1.9%, and erythema 1.5% at mean dose 3.42 mg/24 h. Japanese 603-patient surveillance reported 34.3% adverse drug reactions with site pruritus 10.3%, erythema 7.8%, and dermatitis 5.6%. Patients ≥80 years showed ADR rate 30.4% versus 35.2% in younger groups (P = 0.3778) with similar severity, supporting elderly tolerability (Center for Drug Evaluation, NMPA, 2022; Ito et al., 2022; UCB Pharma, 2021; Zhang et al., 2016; Zhang et al., 2017; Zhu et al., 2025).

Systemic dopaminergic effects—nausea, vomiting, somnolence, dizziness, headache—often emerge during titration, display dose dependence, and frequently attenuate with continued therapy (Center for Drug Evaluation, NMPA, 2022; UCB Pharma, 2021; Zhang et al., 2016; Zhang et al., 2017). Pooled placebo-controlled global data (n = 1,307) classified these as very common (>10%) (Zhu et al., 2025). SP0914 reported nausea 8.9%, dizziness 8.1%, somnolence 8.1%, and vomiting 5.6% versus placebo (Zhang et al., 2016). SP1037 on levodopa background highlighted dyskinesia 10.9% alongside nausea and dizziness, reflecting heightened vulnerability to motor complications in advanced disease. Chinese RWE at lower mean doses showed GI events 4.0% (nausea 2.2%, vomiting 1.2%) and dizziness 1.4%. Japanese RWE reported relatively low hallucination (1.7%) and delusion (0.5%) rates compared with other oral DAs. Impulse control disorder events were rare (binge eating and gambling one case each, 0.2%); one sleep attack (0.2%) was recorded. Labeling warns that sleep attacks and impulse control disorders may occur without warning and impair driving. Immediate patch removal provides rapid exposure reduction—a practical risk-mitigation tool for severe dopaminergic adverse effects.

### 7.2 Application-site reactions and adherence

Although usually mild-to-moderate and non-systemic, ASRs critically influence long-term adherence (UCB Pharma, 2021; Wang et al., 2017). In SP512, ASR was the leading withdrawal reason (∼5% of discontinuations) (Zhang et al., 2016). Japanese prospective data showed 69.7% of patients with ASR-related ADRs ultimately discontinued for local intolerance (Ito et al., 2022). Proactive management converts efficacy into persistence: patients adhering to 14-day site rotation exhibited ASR rate 20.3% versus 50.0% with non-compliant rotation (P = 0.0373) (Zhu et al., 2025). Among patients with ASR wishing to continue therapy, 77.5% achieved symptom control with topical corticosteroids and maintained treatment (Ito et al., 2022). Education on rotating abdomen, flank, buttock, thigh, shoulder, and upper arm—and understanding that mild post-removal erythema from silicone-matrix release reflects transient capillary dilation rather than allergy—reduces anxiety and supports adherence (UCB Pharma, 2021).

Bioavailability varies modestly across recommended sites: minimum 2% difference (upper arm vs. flank) and maximum 46% (shoulder vs. thigh) at steady state, without demonstrated clinically meaningful motor impact. Shoulder application yields highest bioavailability; six-month rotation across lower abdomen, thigh, buttock, flank, shoulder, and upper arm maintained stable mean concentrations. Linear pharmacokinetics within labeled doses support predictable CDS and therapeutic continuity (Center for Drug Evaluation, NMPA, 2022; Geng et al., 2021; Waters, 2013).

Comparative safety positioning versus oral pramipexole and ropinirole in Japanese surveillance suggests favorable neuropsychiatric tolerability profiles, though direct head-to-head randomized comparisons are sparse (Watts et al., 2007). Sleep attacks and impulse control disorders remain class warnings for all DAs; rotigotine is not exempt from structured screening using validated questionnaires and caregiver interview (Ito et al., 2022). The reversibility advantage of transdermal delivery—removing the patch to terminate exposure—contrasts with oral agents whose absorption cannot be halted once ingested (Ito et al., 2022). Pharmacists should incorporate skin examination into routine medication reviews, documenting rotation patterns and prior ASR history to predict persistence risk. Hospital discharge counseling must address heat exposure, MRI safety, and metoclopramide avoidance with the same urgency as dose instructions. A comprehensive summary of the safety and tolerability findings across clinical trials and real-world studies is provided in Table 4.

**TABLE 4:** Safety and tolerability summary of rotigotine transdermal patch.

Domain	Key finding (with references)
Any adverse event (China phase III)	72.5% rotigotine vs. 58.0% placebo (UCB Pharma, 2021 ; Center for Drug Evaluation, NMPA, 2022 (labeling pooled))
Early PD trial (SP0914)	Nausea 8.9%, dizziness 8.1%, somnolence 8.1%, vomiting 5.6% (Zhang et al., 2016 (SP0914))
Advanced PD trial (SP1037)	Dyskinesia 10.9%; nausea, dizziness also reported (Zhang et al., 2017 (SP1037))
China RWE (803 patients)	Overall AE 17.6%; GI AEs 4.0%; site pruritus 6.5% (Zhu et al., 2025)
Japan RWE (603 patients)	ADR 34.3%; site pruritus 10.3%, erythema 7.8%, dermatitis 5.6% ( Ito et al., 2022 )
Elderly (≥80 years)	ADR 30.4% vs. 35.2% (<80 years); *P* = 0.3778 ( Ito et al., 2022 )
ASR and adherence	14-day rotation: ASR 20.3% vs. 50.0% without rotation (*P* = 0.0373) ( Zhu et al., 2025 ; Ito et al., 2022 )
Reversible exposure	Patch removal rapidly lowers drug levels ( UCB Pharma, 2021 )

## 8 Health economics

Health economic analyses distinguish economic value (broad health and resource consequences) from cost-effectiveness, which is typically assessed using the incremental cost-effectiveness ratio (ICER) against willingness-to-pay thresholds. The economic profile of rotigotine includes direct drug costs, quality-adjusted life year (QALY) gains, potential secondary-care savings, caregiver time, and avoided adverse events from treatment interruption. Chinese and international data provide complementary but not fully transferable estimates; local pricing, insurance coverage, and clinical practice patterns ultimately determine payer decisions (Heald et al., 2016; Jiang, 2020; Li et al., 2021).

### 8.1 Economic value

Pharmacoeconomic evaluation balances health gains against resource consumption (Li et al., 2021). For rotigotine, value drivers include symptom control, QoL improvement, and potential reduction in healthcare utilization, offset partly by higher drug acquisition cost (Heald et al., 2016). Cost-effectiveness analyses report QALY gains of 0.0629 versus pramipexole immediate-release and 0.1712 versus pramipexole extended-release, indicating incremental health utility. United Kingdom National Health Service database analyses associated non-oral regimens including rotigotine with reduced unplanned secondary care spending—estimated approximately £718 annual secondary-care savings versus ∼£137 increased drug cost. Once-daily transdermal dosing may also reduce informal caregiving burden. Improved adherence plausibly lowers costs from missed doses and fluctuation-related events, though quantified Chinese evidence remains limited.

### 8.2 Cost-effectiveness analysis

At market prices, Chinese analyses report ICER of 1,118,617.81 CNY per QALY for rotigotine versus pramipexole immediate-release, exceeding the willingness-to-pay threshold adopted in the study (Li et al., 2021). Methodologically, the published Li et al. evaluation and Jiang’s master’s thesis appear to derive from the same Chinese Markov modeling framework (Jiang is a co-author of the journal article) and should be interpreted as linked rather than independent replications (Jiang, 2020; Li et al., 2021). Key model features include a healthcare-payer perspective, long-term Markov health states for PD progression and motor complications, annual discounting of costs and outcomes, price-year inputs anchored to the late-2010s Chinese market, and a willingness-to-pay benchmark tied to multiples of 2018 *per capita* GDP; costs combined drug acquisition with selected medical-resource use, while utilities were drawn from published PD health-state values and mapped clinical endpoints. Under that framework, rotigotine was not cost-effective at list price. Sensitivity analyses identify drug price as the dominant driver: threshold analysis suggests price reductions of approximately 28.5% versus immediate-release and 9.5% versus extended-release pramipexole would approach acceptability thresholds (Li et al., 2021). Updated thresholds, pricing, and insurance policy may alter conclusions. Decision-analytic structures drew clinical parameters from RCTs and cost inputs partly from clinician surveys, introducing potential bias. Interpretation should integrate real-world effectiveness and subgroup context—particularly patients with oral-route failure where rotigotine prevents treatment interruption, aspiration events, or PHS—domains poorly captured in population-average models. Scenario-specific health technology assessment remains a research priority.

Jiang’s pharmacoeconomic thesis and Li et al.'s published Markov model provide the most detailed Chinese cost-effectiveness parameters: incremental QALY gains paired with ICER exceeding three times 2018 *per capita* GDP. Threshold analyses demonstrate that modest price reductions materially shift acceptability, suggesting negotiation and volume-based procurement could alter payer conclusions without requiring new clinical trials. United Kingdom secondary-care analyses by Heald and colleagues associate higher rotigotine and apomorphine prescribing with reduced unplanned secondary expenditures, implying system-level offsets invisible in drug-only budgets. For health technology assessment bodies, scenario analyses should model oral-restricted subgroups separately—perioperative, dysphagia, and enteral-ineligible patients—where rotigotine prevents costly complications including PHS, aspiration pneumonia, and extended rehabilitation stays. Until such stratified models exist, population-average ICERs likely understate value in high-need niches while overstating value for uncomplicated early PD patients well controlled on inexpensive oral generics (Heald et al., 2016; Jiang, 2020; Li et al., 2021).

## 9 Real-world evidence

Randomized trials establish efficacy under controlled conditions but systematically underrepresent elderly multimorbid patients, long treatment horizons, and real-world adherence patterns. Real-world studies of rotigotine address external validity of pivotal trial conclusions and describe how the patch performs when dosing, concomitant medications, and monitoring intensity reflect routine neurology and geriatric practice. Real-world evidence complements trial data and informs treatment recommendations in routine care. Key Chinese and Japanese datasets provide the strongest regional RWE base; European caregiver studies add qualitative dimensions of treatment satisfaction and burden reduction (Ito et al., 2022; Sieb et al., 2015; Zhu et al., 2025).

Real-world studies (RWS) complement RCTs by enrolling broader populations—including elderly and multimorbid patients—and reflecting routine practice constraints (Zhu et al., 2025). Chinese prospective data from 27 hospitals and 803 patients further support the real-world applicability of these findings, while highlighting broader patient-reported and quality-of-life outcomes. Japanese 1-year post-marketing surveillance (603 patients) demonstrated sustained UPDRS III improvement at 3, 6, 9, and 12 months, with approximately 70% achieving mild or greater global improvement and ADL gains. Elderly subgroup analyses (18.6% aged ≥80 years) showed comparable ADR rates (30.4% vs. 35.2%) and motor benefit. Chinese GI adverse event rate was 4.0%. Korean conversion and German CARE-ACT studies highlight once-daily scheduling, feeding independence, and visible dosing as adherence and caregiver facilitators. Collectively, RWE supports the generalizability of findings from controlled studies while underscoring the need for long-term hard outcomes and health-economic follow-up.

Real-world evidence fills critical gaps left by exclusion criteria in pivotal trials (Ito et al., 2022; Zhu et al., 2025). Chinese prospective data at 27 tertiary centers enrolled patients reflective of routine movement-disorder clinic populations, reporting maintenance doses averaging 3.42 mg/24 h—substantially below pivotal trial modal doses—suggesting effective symptom control at lower exposure in practice (Zhu et al., 2025). This dose distribution has implications for both tolerability expectations and budget impact modeling. Japanese one-year surveillance confirms durability beyond 24-week RCT horizons and includes nearly one-fifth of patients aged 80 years or older, supporting extrapolation to China’s aging PD demographic. Korean overnight ropinirole-to-rotigotine conversion demonstrates operational feasibility for outpatient pharmacy services with 72-h follow-up protocols. German CARE-ACT adds stakeholder perspectives often absent from efficacy trials: caregivers and physicians rate patch advantages highest when cognitive impairment or dysphagia complicates oral regimens. Future RWE should capture hospitalization, emergency department visits, falls, and aspiration events to link intermediate clinical outcomes to payer-relevant endpoints.

## 10 Conclusion

Rotigotine transdermal patch is a major non-ergot DA whose clinical distinction lies in 24-h CDS via transdermal delivery rather than conventional oral pulsatile exposure. Broad dopamine receptor affinity and stable pharmacokinetics provide a pharmacological foundation for motor, non-motor, and longitudinal disease management. Evidence from randomized trials, meta-analyses, and real-world studies generally supports the clinical utility of rotigotine for selected patients with PD, although the strength of evidence varies across clinical settings. Independence from gastrointestinal absorption is clinically relevant in perioperative nil per os (NPO) states, dysphagia, gastrointestinal dysfunction, and hepatorenal impairment, although recommendations in these settings should remain proportionate to the predominantly observational evidence base and to the higher vulnerability of advanced patients to dopamine-agonist toxicity. Once-daily application, feeding-independent administration, and visible patch status support adherence and reduce caregiver complexity, including in advanced and elderly populations. Available economic evaluations suggest that rotigotine may be cost-effective in selected settings, although results are sensitive to drug costs, treatment assumptions, and regional healthcare systems.

As PD management evolves from symptomatic control toward long-term individualized care, the role of rotigotine in clinically complex scenarios merits continued evaluation. Structured ASR prevention—with 14-day site rotation and patient education—translates efficacy into persistence. Updated pharmacoeconomic analyses incorporating current pricing, payer policy, and scenario-stratified real-world outcomes will clarify population-level and subgroup value. Integration of rotigotine into perioperative protocols, dysphagia pathways, and end-of-life planning represents a tangible opportunity to align formulation science with patient-centered PD care across the disease continuum (Beckers et al., 2025; Heald et al., 2016; Jiang, 2020; Li et al., 2021; Sieb et al., 2015).

In summary, published evidence supports rotigotine transdermal patch as a clinically differentiated dopamine agonist formulation whose benefit is most pronounced when oral dopaminergic therapy is unreliable, intolerable, or interrupted. By framing the literature around oral-route failure scenarios, integrating Asia/China-specific clinical and economic evidence, and providing pharmacy-oriented conversion and tolerability guidance, this review complements rather than repeats earlier general appraisals such as Frampton (2019). The literature encompasses pharmacokinetic modeling, formulation science, pivotal RCTs (SP0914, SP1037, SP512, RECOVER), meta-analyses, perioperative and dysphagia studies, pharmacoeconomic models, and large Asian real-world cohorts. Optimal use requires appropriate patient selection, evidence-based dose conversion, adherence to 14-day application-site rotation, and integration of patch therapy into perioperative, palliative, and chronic-care pathways. Future research should prioritize updated economic evaluations and prospective registries that quantify clinically meaningful outcomes in populations most likely to benefit from continuous transdermal dopaminergic stimulation. Evidence quality varies by domain: special-population data derive predominantly from observational studies, whereas motor efficacy rests on high-quality RCTs and meta-analyses (Beckers et al., 2025; Li et al., 2021; Watts et al., 2007; Zhang et al., 2016; Zhang et al., 2017; Zhang et al., 2020; Zhu et al., 2025).
